# Supplementary material for: A mouse model of cardiac immunoglobulin light chain amyloidosis reveals insights into tissue accumulation and toxicity of amyloid fibrils
Source: Nat Commun. 2025 Mar 27;16:2992. doi: 10.1038/s41467-025-58307-2 (PMC11950232; doi:10.1038/s41467-025-58307-2)
Supplement: Supplementary file 1 — supplementary information [file 41467_2025_58307_MOESM1_ESM.pdf]

<u>SUPPLEMENTARY INFORMATIONS</u>

Supplementary tables 1-3

Supplementary figures 1-6

Supplemental tables

Table S1: DEG genes between AL- λS-DH and DH mice

Gene	Log FC	FDR	Description
Osbpl3	1.72	0.0007	Encodes one of the members of the Oxysterol-Binding protein family, an intracellular lipid receptor family involved in the maintenance of cholesterol levels in the organism
Snx10	-1.17	0.0083	Encodes a member of the Sortin Nexin family, involved in intracellular trafficking in endosomes through their binding with phosphoinositide
Pyurf	-1.77	0.0023	Encodes for the PIGY Upstream Open Reading Frame, an S-adenosylmethionine-dependent methyltransferase chaperone involved in the stabilization of the coenzyme Q components during its biosynthesis in mitochondria.

Table S2: cardiac and fibrosis-related DEG

	ALvsCtrl		ALvsLS	
	**Log2 FC**	**FDR**	**Log2 FC**	**FDR**
Nmrk2	3.91	0.0007	3.44	0.0062
Irf7	2.09	0.0033	3.45	0.0011
Gpnmb	4.12	1.85E-05	3.47	0.0001
Mmp12	7.6	0.0026	8.17	0.0098
Lgals3	2.7	0.0001	4.09	0.0002
Ltbp2	2.71	0.0029	3.52	0.0058
Timp1	2.63	0.0094	3.74	0.0129
Nppa	3.58	0.0021	1.992	0.095

Table S3: List of antibodies used in the study

Antibody	Reactivity	Clone	Source	Conjugate	Dilution	Applications
Anti-B220	Mouse	Clone RA3-6B2 (Becton Dickinson #562922)	Rat	BV421	1/100	Flow cytometry
Anti-CD138	Mouse	Clone 281-2 (BD Biosciences #558626)	Rat	APC	1/100	Flow cytometry
Anti-human VL6	Human	Monoclonal (from A. Solomon's lab)	Mouse	Unconjugated	1/200	Western-Blot
Anti-lambda	Human	Polyclonal (southernBiotech #2070-01, #2070-04)	Goat	Unconjugated or AP	1/1000	ELISA
Anti-lambda	Human	Polyclonal (Dako #A0194)	Goat	Unconjugated, HRP, gold particles	1/1000	Western Blot
Anti-lambda	Human	Polyclonal (Dako, #F0435)	Rabbit (Fab'2)	FITC	1/2000	Flow cytometry+ IF
Anti-lambda	Human	Clone [4C2] (Abcam, #ab77275)	Mouse	FITC	1/50	IF
Anti-IgG	Rabbit	Polyclonal (Beckman Coulter, #4050-05)	Goat	HRP	1/2000	Western-Blot
Anti-IgG	Mouse	Polyclonal (Beckman Coulter #1031-05)	Goat	HRP	1/2000	Western-Blot
Anti-SAA1+SAA2	Mouse	Polyclonal (Abcam, #ab199030)	Rabbit	Unconjugated	1/120	IF
Anti-ApoA2	Mouse	Polyclonal (AbClonal, #A14690)	Rabbit	Unconjugated	1/100	IF
Anti-IgG	Rabbit	Polyclonal (Molecular probes #A21245)	Goat	AF647	1/100	IF
Anti-SAP	Mouse	Clone 300103, (R&D Systems #MAB2556)	Rat	Unconjugated	1/100	IF
Anti-IgG	Rat	Polyclonal (Molecular probes #A11081)	Goat	AF546	1/100	IF

Supplemental figures

Fig. S1

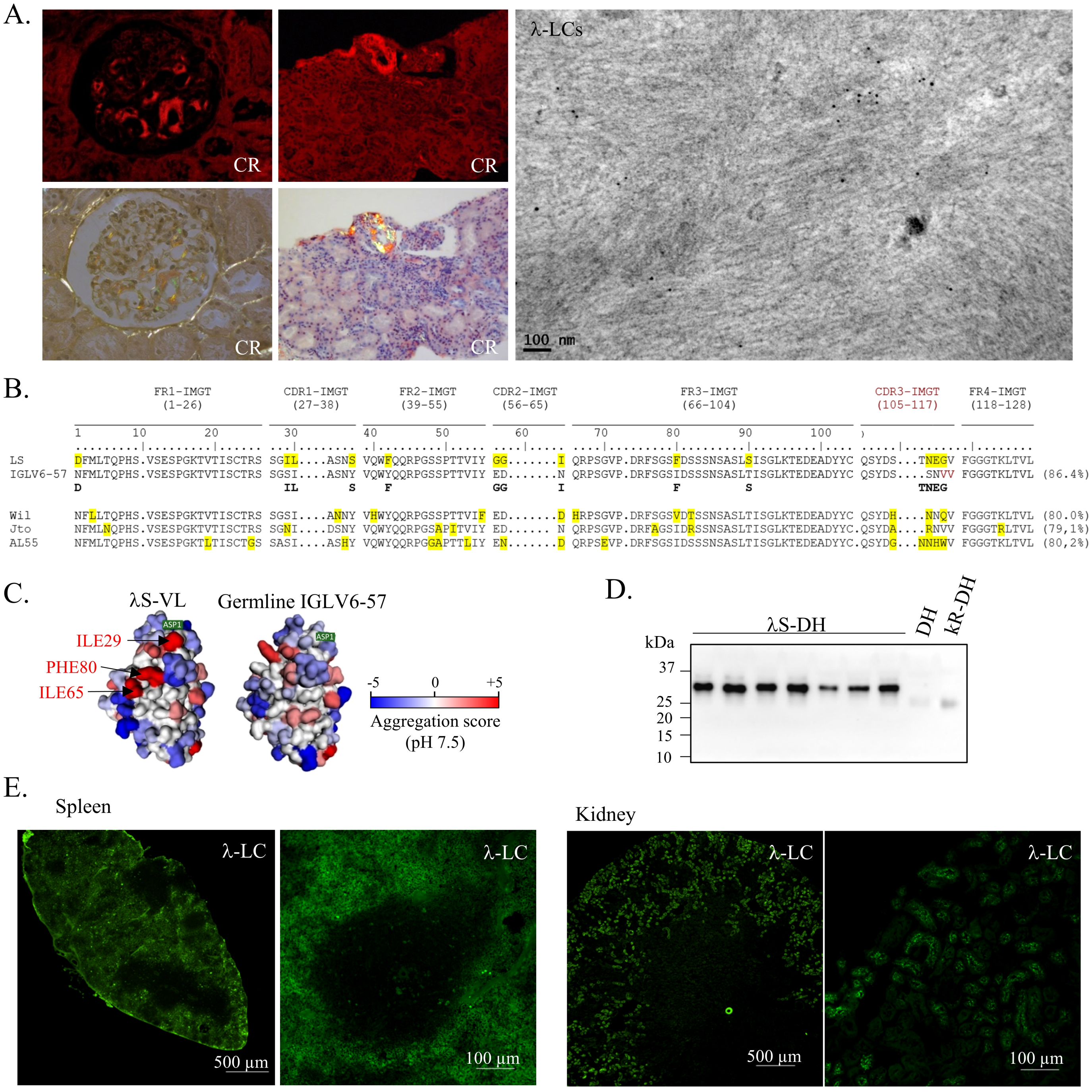

Supplemental figure 1: Characterization of the λS-LC. (A) Kidney biopsy from the patient λS (λS-PT) showing the Congo Red (CR) staining in fluorescence (top left) and its birefringence under polarized light (bottom left) in a glomerulus and in a blood vessel. The characterization of the deposits was performed by immunoelectron microscopy with anti λ-LCs gold-labeled antibody (right). **(B)** Amino acid sequence alignment between the λS-LC (LS) and the germline sequence IGLV6-57 according to the IMGT sequence. Other LCs from the same family were also aligned to our sequence (Wil, Jto and AL55). Mutated amino acids are indicated in yellow, with the percentage of shared sequence is indicated for each one of the alignments. **(C)** *In silico* analysis of the structure of λS-VL sequence with Aggrescan4D software (https://biocomp.chem.uw.edu.pl/a4d/) showed three mutations towards hydrophobic amino acids (Ile29, Ile65 and Phe80) that were absent in the germinal sequence Vλ6-57 (PDB: 2W0K). The green squares correspond to the Asp1 (N-terminal). The aggregation score is given at pH 7.5. **(D)** Confirmation of the presence of the λS-LCs in the serum of our λS-DH transgenic mice (n=7) by Western Blot with an antibody recognizing the human Vλ6-57 LCs, and the absence of this human LC in the serum of controls DH-LMP2A (DH) and κR-DH mice. **(E)** Physiological detection of human λ-LCs in the spleen and the kidney of λS-DH mice (bright green staining), consistent with the production and the reabsorption of LCs, respectively.

Fig. S2

A.

```
                                     Variable Domain

λS-LC     DFMLTQPHSVSESPGKTVTISCTRSSGILASNSVQWFQQRPGSSPTTVIYGGIQRPSGVPDRFSGSFDSSSNSASLSISGLKTEDEADYYCQSYDSTNEGVFGGGTKLTVL
rλS-LC    DFMLTQPHSVSESPGKTVTISCTRSSGILASNSVQWFQQRPGSSPTTVIYGGIQRPSGVPDRFSGSFDSSSNSASLSISGLKTEDEADYYCQSYDSTNEGVFGGGTKLTVL
rλS-VL  STDFMLTQPHSVSESPGKTVTISCTRSSGILASNSVQWFQQRPGSSPTTVIYGGIQRPSGVPDRFSGSFDSSSNSASLSISGLKTEDEADYYCQSYDSTNEGVFGGGTKLTVL

                                     Constant Domain

λS-LC   GQPKAAPSVTLFPPSSEELQANKATLVCLISDFYPGAVTVAWKADSSPVKAGVETTTPSKQSNNKYAASSYLSLTPEQWKSHKSYSCQVTHEGSTVEKTVAPTECS
rλS-LC  GQPKAAPSVTLFPPSSEELQANKATLVCLISDFYPGAVTVAWKADSSPVKAGVETTTPSKQSNNKYAASSYLSLTPEQWKSHKSYSCQVTHEGSTVEKTVAPTECS
rλS-VL  ..........................................................................................................
```

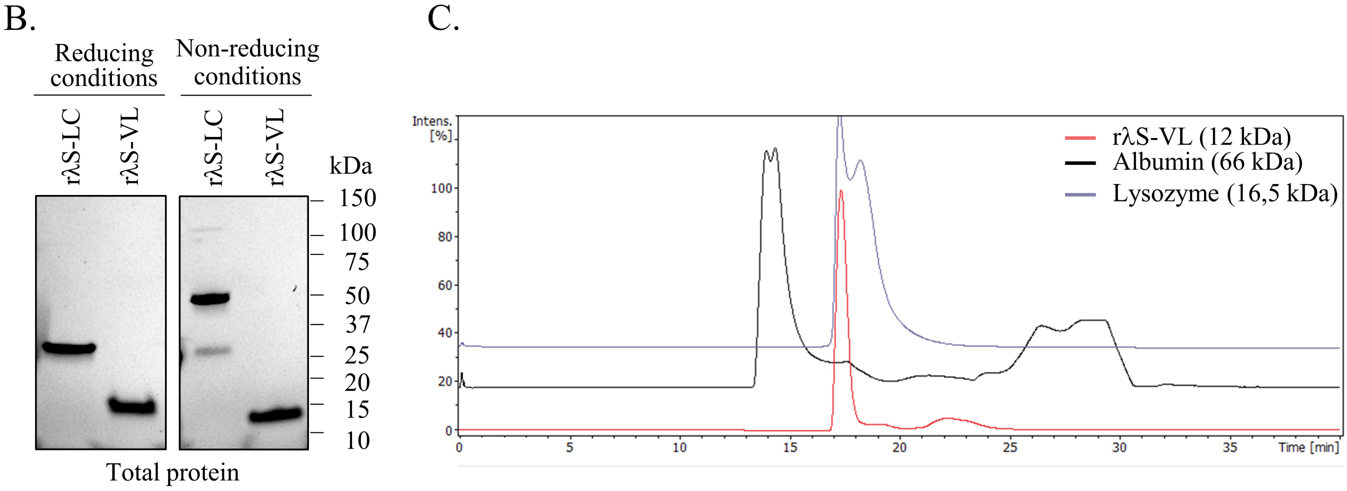

Supplemental figure 2: Analysis of recombinant λS proteins. (A) Alignment of the deduced aminoacid sequences of the patient's λS-LC and the recombinant proteins rλS-LC and rλS-VL. **(B)** SDS-PAGE of the purified recombinant λS proteins, the full-length LC (rλS-LC, ~25kDa) and its variable domain (rλS-VL, ~12kDa) under reducing and non-reducing conditions. Total protein was visualized by the StainFree technology (Biorad). **(C)** Size exclusion chromatography of the rλS-VL confirmed that there were no aggregated forms of the rλS-VL in our samples. Lysozyme (~16.5kDa) and albumin (~66kDa) were used as size standards.

Fig. S3

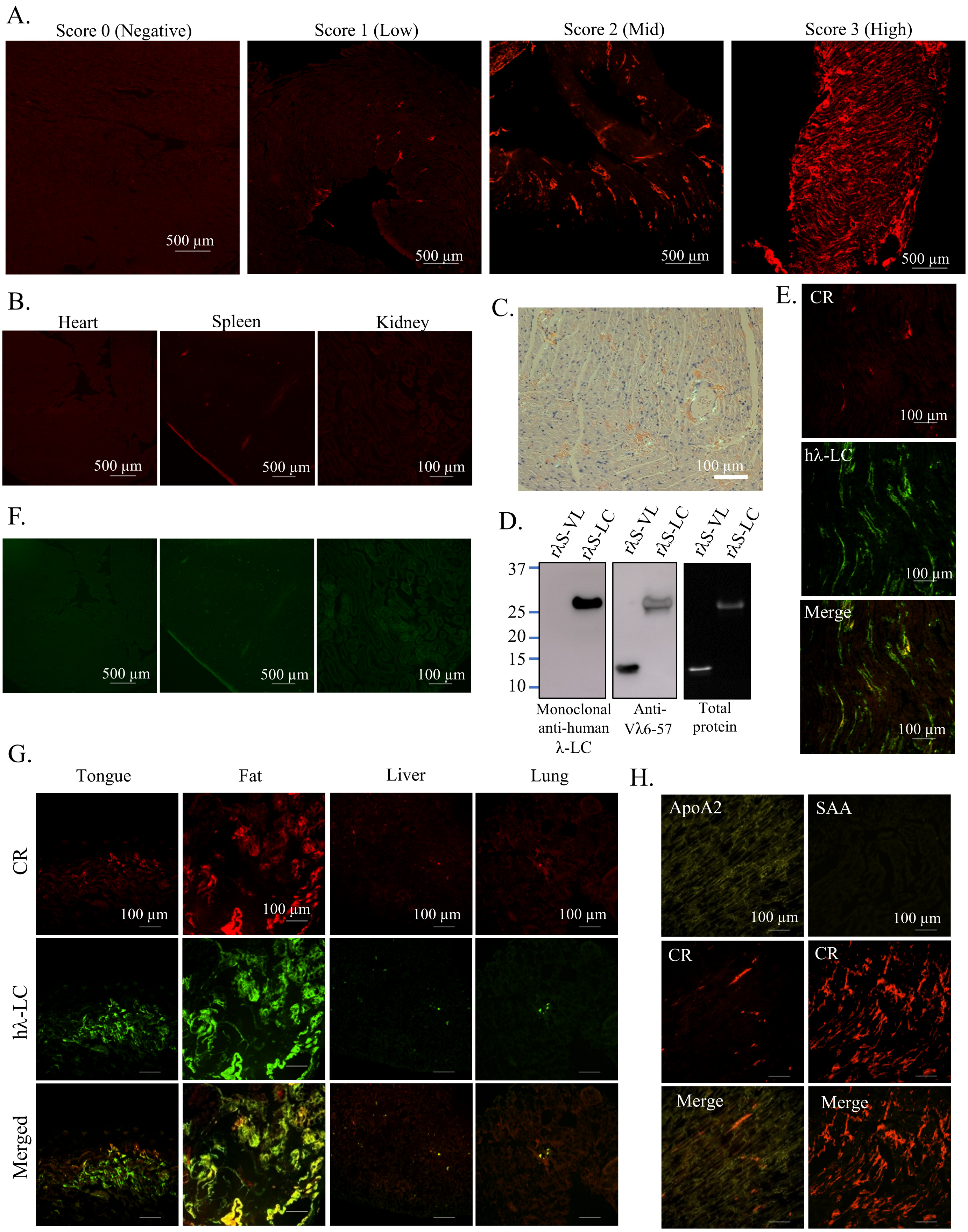
A.
Score 0 (Negative)
Score 1 (Low)
Score 2 (Mid)
Score 3 (High)
500 µm
B.
Heart
Spleen
Kidney
500 µm
100 µm
C.
100 µm
D.
rλS-VL
rλS-LC
37
25
20
15
10
Monoclonal anti-human λ-LC
Anti-Vλ6-57
Total protein
E.
CR
hλ-LC
Merge
100 µm
F.
500 µm
100 µm
G.
Tongue
Fat
Liver
Lung
CR
hλ-LC
Merged
100 µm
H.
ApoA2
SAA
CR
Merge
100 µm

Supplemental figure 3: Amyloid deposits in λS-DH mice. (A) Representative images of the amyloidosis score in the heart of the λS-DH mice according to the CR-stained deposits in fluorescence. "negative", or score 0, corresponds to the total absence of CR staining. "Low", or score 1, corresponds to one of some focal deposits within the myocardium or/and blood vessels. "Mid", or score 2, corresponds to some diffuse deposits within the myocardium. "High", or score 3, corresponds to the diffuse deposits throughout the myocardium. **(B)** Representative image of CR staining in a control mouse (n=29) after induction using the same protocol as for λS-DH mice. **(C)** Hematoxylin-Eosin and CR staining on the heart of a score 3 mouse showing the absence of immune cells infiltration around the deposits. **(D)** Western blot using the monoclonal anti-human λLCs on the recombinant λS proteins (full length rλS-LC and its isolated variable domain rλS-VL) that shows the specificity of this antibody for the constant domain of LCs (left). Western blot with the anti-Vλ6-57 antibody (middle) and the total protein (right) are shown as controls **(E)** CR and the hλ-LC fluorescence in the heart of score 1 λS-DH mice, are colocalized, but more extended hλ-LC can be observed in the intercellular spaces surrounding the CR positive areas. **(F)** hλ-LC staining in mice tissues from (B). **(G)** CR and hλ-LC fluorescence staining in other tissues of λS-DH mice, such as the tongue, liver, fat and lung (n=3). Representative image of a score 2 mouse λS-DH. **(H)** Representative images of Apo-AII (n=2) and SAA (n=5) immunostaining in hearts of a score 1 and score 2 λS-DH mice, respectively.

Fig. S4

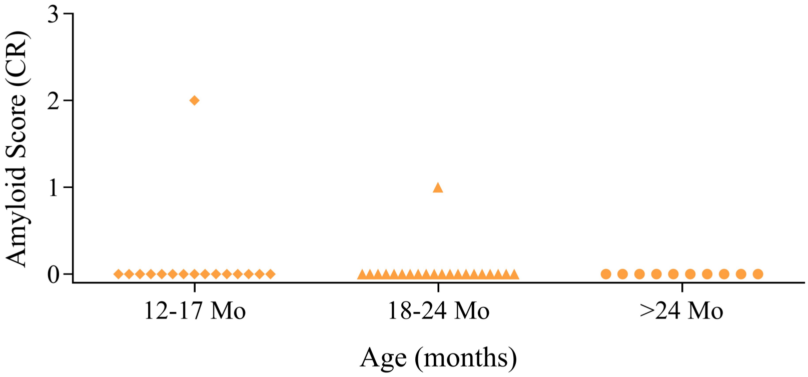

Supplemental figure 4: Rare spontaneous amyloidosis in the λS-DH mice. Spontaneous amyloidosis occurring in the 47-mice cohort analyzed after 12 months (n=16 at 12-18 months (Mo), n=21 at 18-24 months and n=10 at >24 months). Two mice from the cohort developed AL amyloidosis spontaneously at 14 and 18 months. The cardiac score of these mice was evaluated at 2 and 1 respectively.

Fig. S5

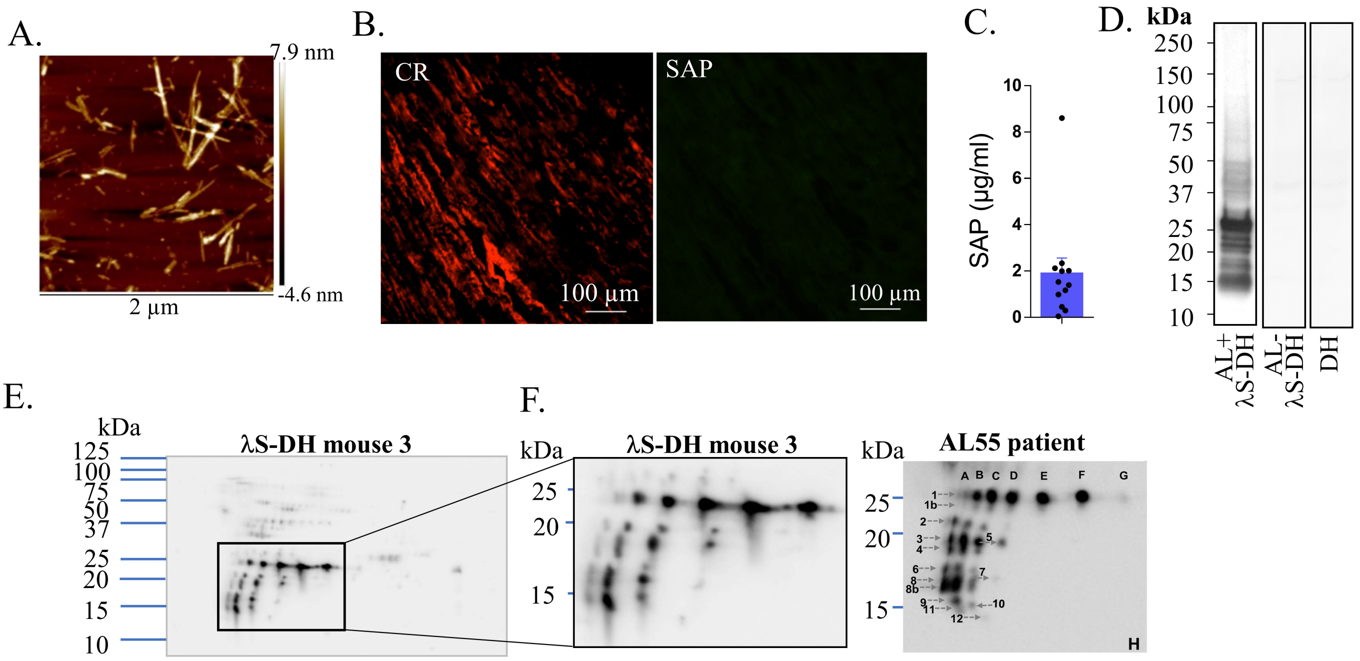

Supplemental figure 5: LC fragmentation patterns in λS-DH mice, control mice and human. (A) Representative Atomic Force Microscopy image of the presence of fibrils samples upon purification with Pras protocol [55] from mice cardiac tissue (n=3). **(B)** Representative images of SAP immunostaining in hearts of AL-positive mice (n=4). **(C)** Serum dosage of the circulating serum amyloid P component (SAP) in the transgenic λS-DH mice (2-6 months old; n=12). Representation of single values with mean (blue bar) ± SD. **(D)** Representative western blotting analysis with a polyclonal antibody against the hλ-LCs of the insoluble material enriched from the heart of amyloid-positive (AL+ λS-DH, n=3) and amyloid-negative (AL- λS-DH, n=1, and DH, n=1) mice. **(E)** Analysis of deposited light chains in an amyloid-positive mouse by 2D-PAGE and 2D western blotting as in (Fig. 5D). The boxed region in mouse 3 matches the region shown in (F). **(F)** 2D western blotting analysis on *ex vivo* fibril-constituting LCs from cardiac tissue extracted from a λS-DH mouse from (E) (left) and, for comparison, an AL patient with an IGLV6-57 LC (AL55) (right). Adapted with permission from Mazzini et al, FEBS, 2021 [12].

Fig. S6

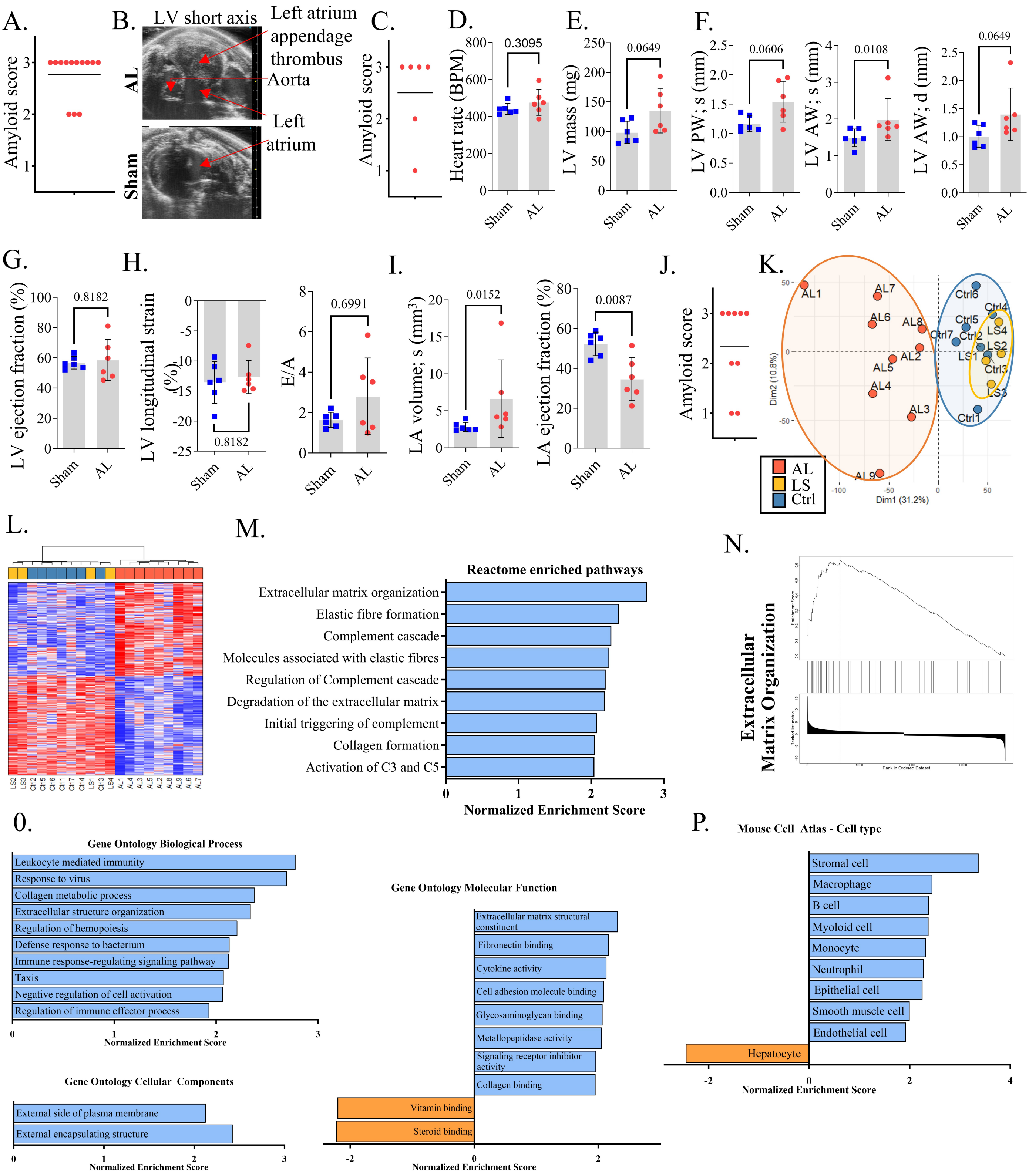

A.
Amyloid score
B.
LV short axis
AL
Sham
Left atrium appendage thrombus
Aorta
Left atrium
C.
Amyloid score
D.
Heart rate (BPM)
0.3095
Sham
AL
E.
LV mass (mg)
0.0649
F.
LV PW; s (mm)
0.0606
LV AW; s (mm)
0.0108
LV AW; d (mm)
0.0649
G.
LV ejection fraction (%)
0.8182
H.
LV longitudinal strain (%)
0.8182
E/A
0.6991
I.
LA volume; s (mm³)
0.0152
LA ejection fraction (%)
0.0087
J.
Amyloid score
K.
Dim1 (31.2%)
Dim2 (10.8%)
AL
LS
Ctrl
L.
M.
Reactome enriched pathways
Extracellular matrix organization
Elastic fibre formation
Complement cascade
Molecules associated with elastic fibres
Regulation of Complement cascade
Degradation of the extracellular matrix
Initial triggering of complement
Collagen formation
Activation of C3 and C5
Normalized Enrichment Score
N.
Extracellular Matrix Organization
O.
Gene Ontology Biological Process
Leukocyte mediated immunity
Response to virus
Collagen metabolic process
Extracellular structure organization
Regulation of hemopoiesis
Defense response to bacterium
Immune response-regulating signaling pathway
Taxis
Negative regulation of cell activation
Regulation of immune effector process
Normalized Enrichment Score
Gene Ontology Cellular Components
External side of plasma membrane
External encapsulating structure
Normalized Enrichment Score
Gene Ontology Molecular Function
Extracellular matrix structural constituent
Fibronectin binding
Cytokine activity
Cell adhesion molecule binding
Glycosaminoglycan binding
Metallopeptidase activity
Signaling receptor inhibitor activity
Collagen binding
Vitamin binding
Steroid binding
Normalized Enrichment Score
P.
Mouse Cell Atlas - Cell type
Stromal cell
Macrophage
B cell
Myoloid cell
Monocyte
Neutrophil
Epithelial cell
Smooth muscle cell
Endothelial cell
Hepatocyte
Normalized Enrichment Score

Supplemental figure 6: Cardiac toxicity analysis. (A) Cardiac scores from the AL+ λS-DH mice analyzed by plasmatic NT-proBNP dosage (n=13). **(B)** 4D-Ultrasound image of the left atrium appendage thrombus in the λS-DH mouse excluded from the analysis. **(C)** Cardiac scores from the AL+ λS-DH mice analyzed by high resolution echocardiography (n=6). **(D)** Heart rates in AL+ λS-DH mice (AL, n=6) and control DH mice (Sham, n=6). **(E)** Calculated left ventricular (LV) mass from control DH (Sham, n=6) and AL+ λS-DH mice (AL, n=6). **(F)** Posterior Wall (PW) LV thickness measured in systole (s) and Anterior Wall (AW) LV thickness measured in systole and diastole (d). **(G)** LV ejection fraction calculated by the longitudinal strain analyzed in parasternal long-axis view. **(H)** Global LV longitudinal peak strain analyzed in parasternal long-axis view and early to late diastolic transmitral flow velocity was given by the ratio E/A. **(I)** Left atrium (LA) volume evaluated at systole and the calculated left atrial ejection fraction. Comparisons in (**D-I**) were performed by Mann-Whitney two-sided test. Histograms show individual values with mean ± SD. **(J)** Cardiac scores from the AL+ λS-DH mice analyzed by RNA sequencing (n=9). **(K)** Principal component analysis showing the distribution of the AL+ λS-DH (AL), AL- λS-DH (LS) and DH (Ctrl) samples analyzed by RNA sequencing according to the variance in gene expression. **(L)** Heatmap showing the 2034 DEG (|log2FC|>1, FDR<0.05) among the 10059 genes analyzed in the comparison between AL (n=9) and both LS and Ctrl groups (n=11). Mice were clustered according to the DE profiles. Red corresponds to overexpressed genes and blue to downregulated genes. Sample colors match with colors in (J). **(M)** Reactome enriched pathways of the DEG analysis from (L). **(N)** Gene Ontology analysis of the DEG according to the biological process (top left), cellular components (bottom left) and molecular function (right). **(O)** Gene Set Enrichment plot representing the distribution of the 41 DEG related to the extracellular matrix organization reactome pathway. **(P)** Mouse Cell Atlas analysis showing the cell type enrichment in the cardiac tissue of the DEG. The represented pathways in (**M, N, O, P**) were significantly enriched (FDR<0.05).
